# Bioprospecting Finds the Toughest Biological Material: Extraordinary Silk from a Giant Riverine Orb Spider

**DOI:** 10.1371/journal.pone.0011234

**Published:** 2010-09-16

**Authors:** Ingi Agnarsson, Matjaž Kuntner, Todd A. Blackledge

**Affiliations:** 1 Department of Biology, Faculty of Natural Sciences, University of Puerto Rico, San Juan, Puerto Rico, United States of America; 2 Institute of Biology, Scientific Research Centre, Slovenian Academy of Sciences and Arts, Ljubljana, Slovenia; 3 Department of Biology and Integrated Bioscience Program, University of Akron, Akron, Ohio, United States of America; Institute of Evolutionary Biology (CSIC-UPF), Spain

## Abstract

**Background:**

Combining high strength and elasticity, spider silks are exceptionally tough, i.e., able to absorb massive kinetic energy before breaking. Spider silk is therefore a model polymer for development of high performance biomimetic fibers. There are over 41.000 described species of spiders, most spinning multiple types of silk. Thus we have available some 200.000+ unique silks that may cover an amazing breadth of material properties. To date, however, silks from only a few tens of species have been characterized, most chosen haphazardly as model organisms (*Nephila*) or simply from researchers' backyards. Are we limited to ‘blindly fishing’ in efforts to discover extraordinary silks? Or, could scientists use ecology to predict which species are likely to spin silks exhibiting exceptional performance properties?

**Methodology:**

We examined the biomechanical properties of silk produced by the remarkable Malagasy ‘Darwin's bark spider’ (*Caerostris darwini*), which we predicted would produce exceptional silk based upon its amazing web. The spider constructs its giant orb web (up to 2.8 m^2^) suspended above streams, rivers, and lakes. It attaches the web to substrates on each riverbank by anchor threads as long as 25 meters. Dragline silk from both *Caerostris* webs and forcibly pulled silk, exhibits an extraordinary combination of high tensile strength and elasticity previously unknown for spider silk. The toughness of forcibly silked fibers averages 350 MJ/m^3^, with some samples reaching 520 MJ/m^3^. Thus, *C. darwini* silk is more than twice tougher than any previously described silk, and over 10 times better than Kevlar®. *Caerostris* capture spiral silk is similarly exceptionally tough.

**Conclusions:**

*Caerostris darwini* produces the toughest known biomaterial. We hypothesize that this extraordinary toughness coevolved with the unusual ecology and web architecture of these spiders, decreasing the likelihood of bridgelines breaking and collapsing the web into the river. This hypothesis predicts that rapid change in material properties of silk co-occurred with ecological shifts within the genus, and can thus be tested by combining material science, behavioral observations, and phylogenetics. Our findings highlight the potential benefits of natural history–informed bioprospecting to discover silks, as well as other materials, with novel and exceptional properties to serve as models in biomimicry.

## Introduction

Spider dragline silk possesses high toughness―the ability to absorb energy before breaking―due to an unusual combination of high tensile strength and elasticity. These properties are typically negatively coupled in synthetic polymers such that spider silk outperforms even high energy absorbing polymers such as Kevlar by ∼300% in terms of toughness [1], [2], [3], [4]. Therefore, spider silk is researched intensively to better understand the interplay between molecular structure and performance, in part hoping to replicate spider silks' desirable properties in biomimetic fibers [2], [5], [6], [7], [8], [9], [10], [11], or even to produce ‘enhanced’ spider silk by infusing it with metals [12]. Equally important, silk also played a critical role in the evolutionary diversification of spiders [13], [14]. In particular, most of the world's 41,000+ species of spiders produce dragline silk from major ampullate silk glands. Dragline silk is used by spiders as lifelines while moving through the environment and to form the supporting frames of most types of prey capture webs. Thus, it is not surprising that the material properties of dragline silk vary among different evolutionary lineages of spiders [10], [15], [16], [17], [18], [19]. For instance, orb weaving spiders spin significantly stronger and tougher dragline silk on average compared to other taxa, which may reflect the importance of the radial threads of orb webs in stopping the tremendous kinetic energy of flying insect prey (Table 1). Yet, even among orb spiders the material properties of dragline silk vary by more than 100%, and across all spiders toughness varies over 20 fold in species examined to date (Table 1, Table S1). A broader understanding of the factors contributing to the mechanical properties of silk therefore clearly requires examination of silks across evolutionarily and ecologically diverse spider lineages. Such bioprospecting of silks promises to reveal novel fibers that have unique combinations of material properties. However, ‘blindly fishing’ for such silks by picking species at random, or systematically sampling all silks of all species are both inefficient. Could scientists instead predict, based upon the natural history and ecologies of different spiders, which species are most likely to spin silks exhibiting exceptional performance properties?

**Table 1:** Biomechanical properties of forcibly silked spider major ampullate silk, ranked from least tough to most tough.

Genus	species	Stiffness (Gpa)	Strength (Mpa)	Exstensibility (ln(mm/mm)	Toughness (MJ/m3)	Guild	Source
*Liphistius*	*murphorium*	5.0	130	0.05	10	wanderer	Swanson et al. 2009*
*Liphistius*	*mayalanus*	3.5	100	0.1	10	wanderer	Swanson et al. 2009*
*Cyclosternum*	*fasciatum*	1.5	50	0.3	10	wanderer	Swanson et al. 2009*
*Grammastola*	*rosea*	1.0	20	0.65	15	wanderer	Swanson et al. 2009*
*Aphenoplema*	*seemani*	1.5	90	0.35	25	wanderer	Swanson et al. 2009*
*Cyroiopagopus*	*pagonus*	3.5	190	0.25	30	wanderer	Swanson et al. 2009*
*Pterinochilus*	*marinus*	2.5	110	0.3	35	wanderer	Swanson et al. 2009*
*Phormictopus*	*cancerides*	3.0	110	0.3	45	wanderer	Swanson et al. 2009*
*Dysdera*	*crocata*	8.0	545	0.177	48	wanderer	Swanson et al. 2006
*Schizocosa*	*mccooki*	4.6	553	0.242	60	wanderer	Swanson et al. 2006
*Poecilotheria*	*regalis*	4.5	210	0.35	70	wanderer	Swanson et al. 2009*
*Hypochilus*	*pococki*	10.9	945	0.17	96	sheet	Swanson et al. 2006
*Agelenopsis*	*aperta*	12.1	958	0.183	101	sheet	Swanson et al. 2006
*Peucetia*	*viridans*	10.1	1089	0.178	108	wanderer	Swanson et al. 2006
*Peucetia*	*viridans*	10.9	1064	0.181	111	wanderer	Agnarsson et al. 2008
*Nephila*	*clavipes*	13.8	1215	0.172	111	orb	Swanson et al. 2006
*Plectreurys*	*tristis*	16.1	829	0.241	112	wanderer	Swanson et al. 2006
*Metaltella*	*simoni*	8.6	765	0.281	114	wanderer	Swanson et al. 2006
*Holocnemus*	*pluchei*	14.3	1244	0.153	115	wanderer	Swanson et al. 2006
*Argiope*	*trifasciata*	9.2	1137	0.215	115	orb	Agnarsson et al. 2008
*Phidippus*	*ardens*	14.2	975	0.189	116	wanderer	Swanson et al. 2006
*Argiope*	*argentata*	8.2	1463	0.184	116	orb	Swanson et al. 2006
*Latrodectus*	*geometricus*	10.2	764	0.310	117	orb	Agnarsson et al. 2008
*Argiope*	*argentata*	5.3	1371	0.214	119	orb	Agnarsson et al. 2008
*Metepeira*	*grandiosa*	10.6	1049	0.235	121	orb	Swanson et al. 2006
*Deinopis*	*spinosa*	10.4	1345	0.191	124	orb	Agnarsson et al. 2008
*Uloborus*	*diversus*	9.1	1078	0.234	129	orb	Swanson et al. 2006
*Latrodectus*	*hesperus*	9.5	959	0.224	132	orb	Agnarsson et al. 2008
*Kukulcania*	*hibernalis*	22.2	1044	0.222	132	sheet	Swanson et al. 2006
*Deinopis*	*spinosa*	13.5	1329	0.185	136	orb	Swanson et al. 2006
*Mastophora*	*hutchinsoni*	9.4	1137	0.268	140	orb	Swanson et al. 2006
*Araneus*	*gemmoides*	8.3	1376	0.224	141	orb	Swanson et al. 2006
*Leucauge*	*venusta*	10.6	1469	0.233	151	orb	Swanson et al. 2006
*Mastophora*	*phryhosoma*	11.3	698	0.340	156	orb	Agnarsson et al. 2008
*Mastophora*	*hutchinsoni*	9.8	1152	0.280	161	orb	Agnarsson et al. 2008
*Araneus*	*gemmoides*	8.6	1414	0.237	164	orb	Agnarsson et al. 2008
*Gasteracantha*	*cancriformis*	8.0	1315	0.301	178	orb	Swanson et al. 2006
*Latrodectus*	*hesperus*	10.2	1441	0.303	181	orb	Swanson et al. 2006
*Gasteracantha*	*cancriformis*	7.3	1199	0.331	193	orb	Agnarsson et al. 2008
*Scytodes*	sp.	10.7	1179	0.357	230	wanderer	Swanson et al. 2006
Average		8.8	878	0.2	107		
**Caerostris**	**darwini**	**11.5**	**1652**	**0.52**	**354** (max **520**)	**orb**	**This study**

*Approximate values estimated from graph.

**Figure 1:**
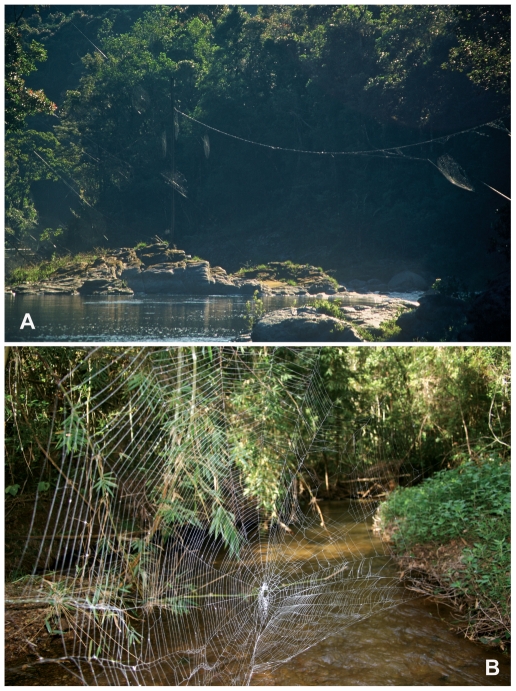
Webs of *C. darwini* spanning streams and rivers. A, several *C. darwini* webs over river in Ranomafana. Individual web area (the extent of the sticky spiral) was about 0.5–1 m^2^, the longest bridgelines exceeded 10 m. B, a web across a small stream in Andasibe-Mantadia NP illustrating architecture. Web width (outermost spirals)  = 105 cm.

Here, we discuss a recent discovery of an exceptional spider web architecture that led us to predict, and subsequently test for, unusual biomechanical properties in the silk of its architect: Darwin's bark spider (*Caerostris darwini* Kuntner and Agnarsson, 2010) [20]. Spiders of the genus *Caerostris* (Araneidae), known in Africa as ‘bark spiders’, are large and eye-catching orbweavers that are widespread throughout the old world tropics. *Caerostris* spiders spin some of the largest orbwebs in nature. Most *Caerostris* build large webs in forest edges or clearings, similar to other exceptionally large orbweavers in related genera and families, such as *Nephila* and some *Araneus*. However, we recently discovered a new riverine *Caerostris* species in Madagascar, *C. darwini*, which displays extraordinary web architecture [20]. In 2001, we first observed giant spider orb webs crisscrossing streams and even large rivers in Ranamofana National Park. We subsequently observed these spiders in Périnet Special Reserve in 2008 and 2010. The large orbs (up to 2.8 m^2^) of *C. darwini* are cast across rivers such that they are suspended directly above the water by bridgelines attached to each riverbank. Length of bridgelines reflects the habitat: across a midsized river in Ranamofana (Fig. 1) most webs had bridgelines between 10 and 14 meters. Across smaller rivers in Périnet bridgelines were much shorter (average 3.5±1.5 m, N = 18), while bridgeline threads reached 25 m (N = 3) across a lake in Périnet (M. Gregorič, pers. comm.). Based upon the large sizes of the orbs as well as the spiders spinning them, and the webs' suspension on such extremely long anchor lines, we predicted that the dragline silk of *C. darwini* would exhibit particularly high performance properties. Here we test the predicted link between the unique web achitecture and the biomechanics of the silk used in the web.

## Methods

### Web measurements and silk collection

Fieldwork took place 29 March-24 April 2008, and 25 February-4 April 2010, in and around the two patches of forest protected by the Andasibe-Mantadia National Park (Périnet Special Reserve and Mantadia forest), in Toamasina Province, eastern Madagascar (altitude 900–1000 m, 18°56′S 48°25′E; 18°47′S 48°25E). Research, collecting, and export permits were obtained from ANGAP (now MNP) and MEFT (permits N° 087/08, N° 088/08, and N° 091N-EA04/MG08), through the ICTE/MICET offices in Stony Brook and Antananarivo. Permits are on file with the senior author. Prior observations on *C. darwini* took place in Ranamofana National Park, Fianantarasoa Province, Madagascar, in 2001 (altitude 1000 m, 21°15′S 47°25′E). In Périnet special reserve we encountered webs of *C. darwini* spun across rivers as previously observed in Ranomafana (see [20], for observations), and across a small lake. We measured sizes of webs for adult females in the field (height, width, distance from hub to top of web, maximum mesh width, number of radii, number of spirals, and height from ground), and then photographed each web for subsequent detailed characterization.

Adult female *C. darwini* were then collected and transported to a greenhouse. Some individuals were simply released to spin on the plants but others were placed within individual frames (described in Agnarsson and Blackledge [21]). Web building took place as soon as a day or up to two months after release in the greenhouse/frames. Some spiders built webs immediately and were preserved after spinning their web. Other spiders were fed live crickets every other day. Orbweavers typically capture all their prey in their web, and it can be difficult to get them to accept prey in the absence of a web. To maximize acceptance of prey in individuals that had not built webs, the hind legs of the crickets were removed, and the cricket wounded to release hemolymph. The cricket was then slowly moved toward the spider, and the hemolymph touched to the spider's mouth. Most individuals accepted prey offered in this manner, and eventually built a web. Silk was sampled from webs within 24 hours after spinning. Dragline silk samples were taken from the radial threads of the orb web while flagelliform silk was collected from the sticky spiral of the web [21], [22], [23], [24]. We also sampled dragline silk “forcibly” by pulling it from restrained spiders as described by [25]. In both cases silk fibers were glued across 16 mm gaps in cardboard mounts using Superglue® (cyanoacrylate), or using Elmer's glue for capture spiral, as described in [26].

### Biomechanical properties

**Figure 2:**
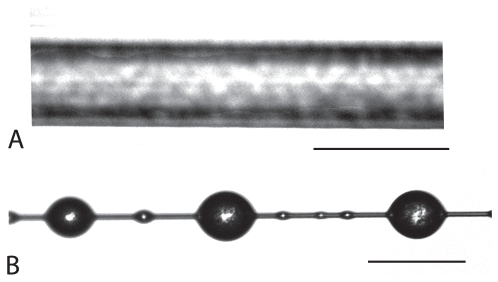
Digital photographs of radial MAP (A) and spiral Flag silk and aggregate silk glue droplets (B). Diameters and volumes of threads and glue are readily measured from these photographs. Scale bars are 10 µm.

The diameter of each silk sample was determined prior to testing by averaging six measurements taken along the length of the fiber from digital photos taken under polarized light microscopy at 1000x (Leica DMLB platform, Fig. 2). Polarization reduces interference around the thin silk fibers while the multiple measurements accounts for the shape anisotropy of some silks [27]. ImagePro 6.2 (Media Cybernetics, Inc.) was used both to take the photographs and to measure the diameters. This method is as accurate as SEM photography, but does a better job of accounting for intra-individual variation in silk diameter as it allows measurement of the diameters of each silk sample tested [27]. The silk fibers were then fastened to the grips of a Nano Bionix tensile tester (Agilent Technologies, Oakridge, TN) and pulled until failure at an extension of 10%/sec at a resolution of 0.1 µm. The resulting force values were recorded to a resolution of ∼1 µN.

For each sample, we calculated four mechanical properties from the force-extension curves. (1) Ultimate strength (true breaking stress) is the force required to break a fiber relative to the instantaneous cross sectional area of the fiber, the latter calculated using an assumption of constant volume during extension [28]. (2) Extensibility (true breaking strain) is the natural log of the breaking length divided by original length. The standard isovolumetric assumption was applied [29]. (3) Initial stiffness (Young's modulus) is the slope of the stress–strain curve over the initial elastic region. (4) Toughness is the energy absorbed by a fiber prior to rupture, calculated as the area under the stress–strain curve divided by sample volume (initial cross-sectional x length of the fiber).

## Results

### Web architecture

*Caerostris darwini* was the most common *Caerostris* species in Périnet, and unlike related *Caerostris* species, occurred almost exclusively along, and in most cases across, streams, rivers, and lakes. The webs of *C. darwini* (Fig. 1; N = 18) were quite different from other *Caerostris* webs known to us in that they were “less dense” – webs were supported by relatively few radii (mean±SD = 21.8±4.0, range 17–25) compared to other *Caerostris* species in the area (pers. obs.), and even to most araneids and nephilids (Table 2). Some webs contained a few secondary radii, radial threads that do not go all the way to the center of the web that are present also in some other giant orbwebs, such as *Nephila* and *Nephilengys* [30], [31]. Typical spacing between spirals was about 0.6 cm, with maximum mesh with ranging from 0.8–3.5 cm. These webs are therefore relatively open meshed compared to typical orbweavers (Table 2, where only *Nuctenea* has a sparser web), in particular compared to other giant webs such as those spun by *Nephila* who uses a very high number of secondary radii to make an extremely dense web. *Caerostris darwini* webs in the field ranged from 1.900–28.000 cm^2^ in size (area of capture spiral), with bridgelines extending up to 25 m (Fig. 1, Table 2). Many webs showed conspicuous signs of damage and repair, others had large open holes that were present on consecutive days. Both observations suggest that webs are maintained for several days. In contrast, most terrestrial orbweavers remove and reconstruct webs daily. In the lab, *C. darwini* produced much smaller webs averaging 800 cm^2^, nevertheless, as seen in the field, webs in captivity were still relatively sparse with large mesh widths and an average of only 17 radii.

**Table 2:** Orb web architectures from lab from the range of orbweavers examined by Sensenig, et al. [39], and from large orbweavers found in the field by the authors.

Species	Radii #	Mesh width mm	Size cm^2^	N	From	Comments
*Uloborus glomosus*	24	0.55	42	?	lab	
*Cyclosa conica*	34	0.16	89	5	lab	
*Mangora maculata*	45	0.09	108	9	lab	
*Metepeira labyrinthea*	25	0.23	145	9	lab	
*Araneus marmoreus*	18	0.27	147	5	lab	
*Zygiella x-notata*	29	0.20	165	8	lab	
*Micrathena gracilis*	49	0.14	211	15	lab	
*Araneus trifolium*	18	0.47	294	7	lab	
*Neoscona arabesca*	25	0.28	295	15	lab	
*Lecauge venusta*	28	0.25	312	12	lab	
*Araneus diadematus*	31	0.22	357	4	lab	
*Argiope trifasciata*	37	0.29	377	9	lab	
*Gasteracantha cancriformis*	30	0.27	425	11	lab	
*Argiope aurantia*	25	0.48	435	17	lab	
*Tetragnatha*	19	0.37	458	4	lab	
*Nuctenea umbratica*	17	0.75	474	7	lab	
*Nephila clavipes*	45	0.32	609	13	lab	
*Verrucosa arenata*	19	0.50	656	8	lab	
*Larinioides cornutus*	18	0.56	691	26	lab	
*Neoscona domiciliorum*	26	0.34	734	4	lab	
*Neoscona crucifera*	24	0.36	853	5	lab	
*Eustala* sp.	27	0.35	934	3	lab	
*Herennia multipuncta*	22*	na	1449	4	field	20 primary radii
*Herennia etruscilla*	30*	na	1678	2	field	20 primary radii
*Nephila ardentipes*	120*	na	2335	17	field	30 primary radii
*Nephilengys malabarensis*	60*	na	3004	7	field	17 primary radii
*Nephila inaurata*	100*	na	3244	10	field	25 primary radii
*Nephila pilipes*	195*	na	3615	19	field	38 primary radii
*Nephilengys borbonica*	29*	na	4060	10	field	16 primary radii
Average	42	0.34	972			
**Caerostris darwini**	**22**	**0.60**	1900–28000	∼20	field	
**Caerostris darwini**	17	0.49	793	7	lab	

*total radii and primary radii, estimated from photographs M. Kuntner.

na = not available.

#### Silk biomechanics

**Figure 3:**
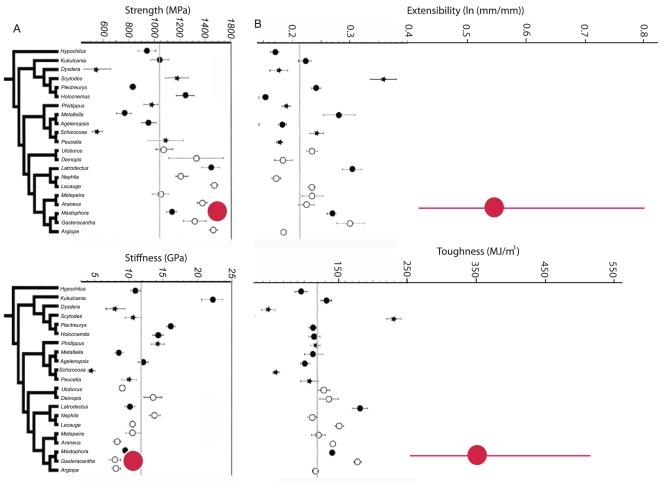
Tensile performance of *C. darwini* dragline silk compared to other spiders. A) the strength of *Caerostris* silk is high but unexceptional while stiffness is slightly below average. B) In contrast extensibility and toughness of *C. darwini* forcibly pulled silk both far surpass that found in the broad taxonomic sample by Swanson et al. (2007). Red lines show the range of *Caerostris* silks with dots indicating average values. Note that *Caerostris* was not included in the phylogeny of Swanson et al. (2007), red dots are placed arbitrarily among other orbweavers. Vertical grey lines show average values across the spiders examined by Swanson et al. (2007).

**Figure 4:**
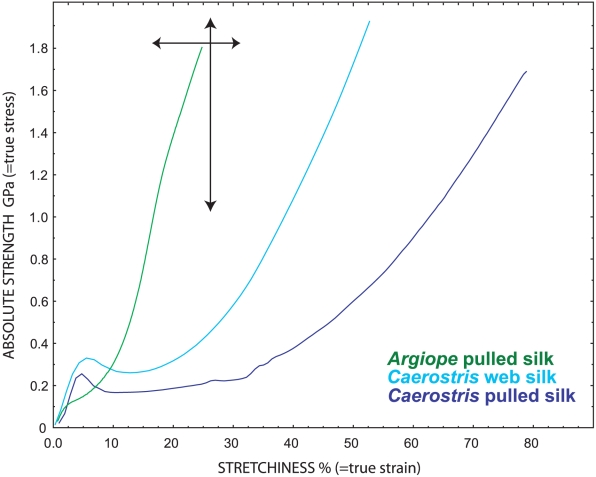
Comparison of *C. darwini* silk tensile tests to other orb spiders. Each line represents a single silk sample, with strength measured as stress on the y axis and stretchiness on the x axis. The tensile strength and total stretchiness of a silk are represented by the end of the lines, where the fibers break. The curves for *Argiope* are typical for orb weaving spiders, while the bars represent the total variation in pulled dragline silk strength and stretchiness across spiders (from Swanson et al 2006). *Caerostris darwini* silk is far stretchier than typical major ampullate silk. This allows *Caerostris* silk to absorb massive amounts of kinetic energy without breaking, making it the toughest biological material ever discovered. Across all spiders, Swanson et al (2006) found that silk toughness ranged from 50–230 MJ/m3. These values are already exceptional when compared to synthetic materials like Kevlar (33 MJ/m3). However, silk from *C. darwini* exhibited an average toughness of 350 MJ/m3 across 10 spiders, with some individuals ranging up to 500 MJ/m3.

Major ampullate (radial thread) silk from webs exhibited ultimate strength (true breaking stress: mean±SD) of 1850±340 MPa, extensibility (true breaking strain) 0.33±0.08 mm/mm, initial stiffness (Young's modulus) 11.5±5.1 GPa, and toughness 27±68.6 J/cm^3^ (Figs. 3–4). The web silk is tougher than any of the silks tested in two broad taxonomic surveys by Swanson et al [15] and by Sensenig et al. [39] (Table 1, Table S1).

Major ampullate forcibly silked threads had ultimate strength (true breaking stress) of 1652±208 MPa, extensibility (true breaking strain) 0.52±0.22 mm/mm, initial stiffness (Young's modulus) 11.5±2.6 GPa, and toughness 354±93 J/cm^3^ (range 233–520 J/cm^3^) (Figs. 3–4). While highly variable, maximum extensibility for pulled major ampullate silk was 0.91, a truly exceptional stretchiness for this kind of silk. Strain and toughness, especially of pulled silk, outperform all previously described silks (Table 1, Table S1).

Capture spiral thread silk from web exhibited ultimate strength of 1400±423 MPa, extensibility 1.01±0.14, and toughness 270±91.4 J/cm^3^.

## Discussion

Bioprospecting promises to find exceptional biological materials more rapidly than random sampling. By combining fields as diverse as natural history, ecology, taxonomy, behavior and biomaterial science we here unravel the biomechanical properties of major ampullate silk from Darwin's bark spider (*Caerostris darwini*), a giant Malagasy riverine orb weaving spider, which was only recently discovered during our expeditions [20]. To our knowledge, *C. darwini* produces the toughest biological material examined to date (Table 1, Table S1):

*Caerostris darwini* major ampullate silk is, on average, about twice as tough as typical silks spun by other orbweavers (Figs 3–4, Table 1, Table S1). Spider dragline silk is already deservedly renowned for its high toughness of ∼150 MJm∧-3, which outperforms both steel and Kevlar [32]. Yet, *C. darwini* silk is far higher performing, absorbing about ten times more kinetic energy before fracturing than does Kevlar. The major difference between *Caerostris* silk and silk of other orbweavers lies not in stiffness, where *Caerostris* silk is average, nor in its strength, though it ranks among the stronger spider silks (Table 1, Table S1). Instead, the elasticity of *Caerostris* silk is approximately twice that of other orb spiders' dragline silk. This extreme extensibility combines with its high strength to explain the silk's incredible toughness. Interestingly, *C. darwini* spiral silk is extremely tough as a result of increased strength while maintaining normal sticky silk stretchiness.

For radial silk, elasticity is also the most variable material property we measured in *Caerostris* silk. Of the 30 tested fibers, elasticity (true strain) ranged from fairly typical silk elasticity at 0.21 mm/mm, to a truly exceptional 0.91 – the latter value approaching the elastic spiral silk of orb webs. We obtained some of the dragline threads by forcible silking – pulling the threads directly from the major ampullate spigots of restrained spiders. Such silk is normally stiffer and less extensible than silk in orb webs because the spiders resist forcible silking by applying greater shear stress in the spigot [33]. Yet, forcibly silked fibers from *C. darwini* were more extensible than native dragline silk from the web. Even the least elastic forcibly silked fibers were stretchier than the major ampullate silk of a diverse sampling of spiders [15]. These observations suggest that understanding the molecular basis of the extensibility of *C. darwini* silk is key. Reduced crosslinking between silk proteins within the amorphous regions of the silk fibers, decreased orientation of molecules, or even novel secondary structures could all explain the silk's high extensibility, but such speculation remains to be tested.

While our discovery represents an important increase in the known performance of natural spider silks, we believe it possible, and perhaps even likely, that even better performing spider silks remain to be discovered. Currently known spider dragline silks range in toughness from 10–520 J/cm^3^, yet they represent only a tiny fraction of the known phylogenetic and ecological diversity of spiders. Therefore, the actual range in performance is probably much greater. Also, dragline silk is classically considered the toughest and strongest of the diverse toolkit of different silks spun by spiders, but this is not necessarily the case. In the first description of the molecular sequence and mechanical properties of aciniform prey wrapping silks, Hayashi et al. [34] found that it exhibited toughness of about 370 J/cm^3^, far greater than any known silk, until now. Furthermore, the measured toughness of fibers depends on testing conditions, such as strain rate. All our tests were performed at strain rates common in biomechanical research, but relatively slow compared to how silks function in nature, when stretched suddenly by flying insects. Gosline et al. [4] showed that under very high strain rates (failure in about 0.02 s, strain rate of 30 s^−1^) *Araneus diadematus* dragline silk (toughness 193 J/cm^3^ under strain rates we used) could reach an astronomical toughness of up to 1000 J/cm^3^. Thus testing more types of *C. darwini* silks and under a variety of strain rates will likely reveal an even higher maximum toughness of its silk fibers.

Our findings have implications for the development of biomimetic fibers. Depending on the desired qualities of the biomimetic fibers, work could profitably focus on particular species of spiders that have maximal performance in those properties, rather than mimicking simply the properties of the species that happen to be best studied. We found exceptionally tough silk associated with unusually large webs and long bridgelines. Similarly, the most adhesive silk discovered to date comes from a spider with a very unusual, highly reduced web architecture (*Hyptiotes*, [35]), suggesting that web architecture may offer a clue to promising species for bioprospecting. Recent work on spider silk biomechanics has broadened to include more taxa than the two to three species of orb spiders that were the exclusive focus of most early studies [6], [15], [19], [21], [22], [36], [37]. Our findings highlight the potential benefits of utilizing knowledge of spider biology to bioprospect among more than 41.000 spider species to discover fibers exhibiting particularly high performance. *Caerostris darwini* builds some of the largest orb webs known among spiders and constructs longer bridgelines than other giant orbweavers that we are aware of, spanning across even large rivers [20]. Although some other spiders build webs above the edges of streams and rivers, or even directly attached to water [38], *C. darwini* is distinguished by spanning such large bodies of water with its webs. This striking architectural feat allows *C. darwini* to spin webs in a unique microhabitat thereby gaining access to prey in the air column above large rivers. How individual spiders of Malagasy *Caerostris* achieve the unique architectural feat of casting webs across entire rivers is as yet unknown. But, it likely involves bridging, a behavior where strands of silk are released into the air and drift until entangling a distant substrate, thus establishing initial bridgelines. We are currently testing this speculative hypothesis, but the great distances involved suggest that *C. darwini* must require more time and effort to establish webs, compared to species in terrestrial habitats, where bridging points are closer. Indeed, many orb spiders readily change the locations of webs, re-spinning them daily. In contrast, the persistence of identifiable patterns of web damage that persists over 24 hrs suggests that *C. darwini* webs are at least sometimes longer lasting. Also, the structural threads must sustain relatively high constant loads without greatly sagging. Not only must these threads support the very large web and heavy spider, but they must do so while attached to moving branches and exposed to wind and rain in the unsheltered river valley. Clearly, it is advantageous to anchor the webs using especially tough bridgelines to maximize the life of the web, the critical connection established across river, and to reduce the likelihood of the web collapsing into the river. We found indirect evidence that webs are long-lasting. The capture areas of webs may be rebuilt almost daily, but many webs showed clear evidence of ‘wear and tear’ with large holes and other structural damage, that were not repaired immediately. Furthermore, spiders remained for at least several weeks in the same site, relying on long lasting bridgelines. Silk that is able to absorb large amounts of energy before breaking would be optimal in these circumstances. Finally, it is worth noting that two other species that often build long bridgelines, *Gasteracantha* and *Verrucosa* (pers. obs.), also have exceptionally tough dragline silk (Table 1, Table S1).

Given the high toughness of *C. darwini* silk, one may wonder if it could be a result of experimental error. However, we feel confident that this is not the case. First, each of the six individual spiders from which fibers were tested exhibited average toughness values exceeding 250 J/cm^3^, despite differences among individual spiders in size, condition, thickness of fibers and other factors. Second, there is no evidence that the sizes of silk threads were underestimated. Other, size-dependent performance parameters (e.g. stiffness and tensile strength) are unexceptional. Moreover, the relatively large diameters of the fibers are easily measured using established protocols. Third, *Caerostris* silk was tested during a year-long series of experiments on fibers of over 20 species of orbweavers. The researchers had accumulated much experience and the performance of silk from other spiders tested during the same time period was consistent with previous data.

The rivers across which adult *C. darwini* suspend their webs are used as flyways by large insects, birds and bats. It is tempting to speculate that *Caerostris* evolved giant webs under selection to capture such extraordinarily large prey. While the extreme toughness of *C. darwini* silk is consistent with this hypothesis, the most commonly observed prey items in the field were relatively small. Prey included bees and small dragonflies, while a major prey catching event involved several webs full of aquatic insects (Mayflies - Ephemeroptera) [20]. Field observations are clearly needed to determine how *Caerostris* utilize these remarkable webs. However, as appealing as the hypothesis that *Caerostris* evolved such extraordinary silk under natural selection for the capture of giant prey might be, the meager preliminary data that exist on *Caerostris* prey only found abundant smaller aquatic insects in their webs [20]. Regardless, the webs allow access to habitat and prey that other spiders cannot utilize.

In summary, we hypothesize that the world's longest orb-webs and toughest silk coevolved within the genus *Caerostris* as species began to occupy a novel habitat – the flyways above rivers. This spectacular adaptation puts *C. darwini* at a considerable advantage over other forest dwelling species of orb spiders, by allowing *Caerostris* to ensnare abundant insects or other prey flying over water. Perhaps most importantly, our study demonstrates how understanding the ecologies of spiders can play a critical role in biomimicry. The discovery of *C. darwini's* incredibly tough dragline silk followed from our initial observations about its extraordinary orb webs and greatly expand our understanding of the potential performance of silk fibers.

## Supporting Information

Table S1 Biomechanical properties of spider major ampullate silk collected from web radii, ranked from toughest to least tough (table above). Biomechanical properties of some other natural fibers and of some high performance man made fibers (table below).
(0.01 MB XLS)
